# Editorial: AI/ML in pharmacovigilance and pharmacoepidemiology

**DOI:** 10.3389/fdsfr.2024.1517365

**Published:** 2024-11-13

**Authors:** Wen Zou, Pantelis Natsiavas, Assaf Gottlieb

**Affiliations:** ^1^ Division of Bioinformatics and Biostatistics, National Center for Toxicological Research, U.S. FDA, Jefferson, AR, United States; ^2^ Institute of Applied Biosciences, Centre for Research and Technology Hellas (INAB|CERTH), Thessaloniki, Greece; ^3^ McWilliams School of Biomedical Informatics, University of Texas Health Science Center at Houston, Houston, TX, United States

**Keywords:** artificial intelligence, machine learning, pharmacovigilance, pharmacoepidemiology, AI trends

This Research Topic of papers corresponding to Frontiers’ Research Topic on “AI/ML in Pharmacovigilance and Pharmacoepidemiology” illustrates methods of Artificial Intelligence/Machine Learning (AI/ML) solutions being investigated or applied to the domain of pharmacovigilance (PV) and pharmacoepidemiology (PE). There is widespread excitement about the use of AI across multiple scientific/technical/industry/government domains, including PV/PE. This Research Topic highlights different aspects in which AI/ML is applied in these domains. Here, we highlight the different aspects covered within this Research Topic and provide the editors’ forecast for the future of AI/ML in the field.

The opportunities and challenges presented by AI in PV and PE were reviewed by Crisafulli et al. They describe the potential for utilizing real-world data (RWD) sources to improve clinical practice in drug discovery and repurposing, clinical trials, and the development of digital therapeutics. They further discuss challenges of implementing AI in pharmacological management, including the interpretability and transparency of AI systems, enabling continuous learning and adaptation, integrating AI with existing healthcare IT infrastructures, and enhancing digital literacy among healthcare professionals and patients.

Other papers in this Research Topic cover different aspects of using AI for PV, informing the development of safe and effective drugs. Wanika et al. analyzed data from multiple clinical trial studies and utilized ML algorithms to identify 12 groups of Small Cell Lung Cancer patients who are at risk of developing common serious adverse events, which could aid in specialized monitoring and management of at-risk patients (Wanika et al.). Liu et al. used ML to evaluate the predictive values of drug targets, drug classification (i.e., level 2 ATC codes), and protein-protein interaction networks for prediction of frequently-occurring drug side effects (Liu and Wilson). Li et al. evaluated the performance of three large language models (LLMs) in automating literature screening for pharmacovigilance (Li et al.). They found that with properly designed prompts, the LLMs achieved high sensitivity and reproducibility, indicating their potential to efficiently identify relevant articles and filter out a significant number of irrelevant articles. Finally, Kassandros et al. identified factors influencing the acceptance and market penetration of generics in Greece through ML models (Kassandros et al.).

We identified four key trends emerging from the papers included in this Research Topic and related works (Bate and Luo, 2022; Kassekert et al., 2022):

- LLMs and Natural Language Processing are the primary AI methodology likely to be widely adopted in the near future, owing to the broad availability of commercial services (e.g., ChatGPT). These models offer scalability in handling the vast amount of unstructured free-text data available across multiple PE/PV use cases.
- While AI could support complex PE/PV tasks (e.g., signal detection), the immediate uses of AI would likely put an emphasis on automating “simpler” tasks which still require significant manpower, e.g., Individual Case Safety Reports’ (ICSRs) deduplication, triage and others.
- Fully exploiting the prospects of AI requires “multiple modalities” of data processing, i.e., the combined “reasoning” upon various kinds of data (integration of ICSRs, biochemical data, signaling pathways, RWD from healthcare, social media data, lifestyle data, etc.) and the combined use of various computational approaches/algorithms. This is a key issue which will pose challenges for adopting AI in scale for PV/PE as this would also require the development and validation of tools that combine these multiple modalities.
- While there is certainly a great deal of enthusiasm surrounding ML right now, “hybrid AI” i.e., the combination of symbolic AI approaches (e.g., the use of ontologies and automatic reasoning upon them) with ML could be one of the future technical paradigms to play a key role in PV and PE. The integration of symbolic knowledge structures could support valuable experts’ knowledge to ML approaches and facilitate heterogeneous ML algorithms’ results’ integration improving the overall outcomes. Furthermore, the use of well-defined human understandable knowledge structures could increase the outcomes’ explainability, also playing a key role towards the adoption of AI-based systems.

Along these lines, it should be noted that regulatory organizations have a key role in both regulating the use of AI for PV/PE and supporting the research and development of computational approaches and relevant data infrastructures. For example, the European Medicines Agency (EMA) has recently setup an infrastructure to enhance the processing of RWD significantly boosting interest in the use of RWD for the pharmaceutical industry. US Food and Drug Administration (FDA) has been broadly exploring the application of AI/ML in PV, with one of the focuses on AI application aimed at processing and evaluation of ICSRs submitted to the FDA Adverse Event Reporting System (FAERS) (Ball and Dal Pan, 2022).

Emerging technologies, such as AI, are evolving rapidly. What we understand today may change in the very near future. The development of LLMs, AI and ML are of great interest for applications in PV and PE designed to improve the efficiency of managing ever-increasing volumes of safety data. AI/ML is expected to significantly enhance PV by automating and improving drug safety monitoring processes. AI/ML models will potentially enable real-time, continuous monitoring of drug safety, reducing the lag time between adverse event occurrence and reporting. AI is also expected to facilitate better risk stratification, personalizing drug safety measures based on individual genetic profiles, comorbidities, and drug interactions.

In the coming years, we expect more sophisticated AI tools to be integrated into regulatory processes, helping regulators and healthcare providers make more informed decisions about drug approvals, withdrawals, and safety warnings. It’s worth noting that although AI is promising and can play a role in PV, human expertise remains essential. While human oversight will remain critical to ensuring ethical and accurate AI implementation, AI-driven PV will undoubtedly contribute to more proactive and efficient drug safety practices.

As a whole, we anticipate that the use of AI to support PV/PE will remain an active research and development domain for the coming years and we hope that this Research Topic, published by Frontiers, provides an important stepping-stone in this journey.
